# The Reversible Carnitine Palmitoyltransferase 1 Inhibitor (Teglicar) Ameliorates the Neurodegenerative Phenotype in a Drosophila Huntington’s Disease Model by Acting on the Expression of Carnitine-Related Genes

**DOI:** 10.3390/molecules27103125

**Published:** 2022-05-13

**Authors:** Carla Bertapelle, Maria Rosaria Carillo, Nunzio Antonio Cacciola, Yulii V. Shidlovskii, Gianfranco Peluso, Filomena Anna Digilio

**Affiliations:** 1 Research Institute on Terrestrial Ecosystems (IRET), UOS Naples-CNR, Via Pietro Castellino 111, 80131 Naples, Italy; carla.bertapelle@unina.it (C.B.); mariarosaria.carillo@unicampania.it (M.R.C.); gianfranco.peluso@cnr.it (G.P.); 2 Department of Neurosciences, Reproductive and Odontostomatological Sciences, University of Naples Federico II, Via Pansini 5, 80131 Naples, Italy; 3 Department of Experimental Medicine, University of Campania “Luigi Vanvitelli”, Via Santa Maria di Costantinopoli 16, 80138 Naples, Italy; 4 Department of Veterinary Medicine and Animal Productions, University of Naples Federico II, Via Pansini 5, 80131 Naples, Italy; 5 Department of Gene Expression Regulation in Development, Institute of Gene Biology, Russian Academy of Sciences, 34/5 Vavilov St., 119334 Moscow, Russia; yul.biogen@gmail.com; 6 Department of Biology and General Genetics, I.M. Sechenov First Moscow State Medical University, 8, bldg. 2 Trubetskaya St., 119048 Moscow, Russia

**Keywords:** *Drosophila melanogaster*, Huntington’s disease, neurodegeneration, L-carnitine, carnitine shuttle, mitochondria, β-oxidation

## Abstract

Huntington’s disease (HD) is a dramatic neurodegenerative disorder caused by the abnormal expansion of a CAG triplet in the huntingtin gene, producing an abnormal protein. As it leads to the death of neurons in the cerebral cortex, the patients primarily present with neurological symptoms, but recently metabolic changes resulting from mitochondrial dysfunction have been identified as novel pathological features. The carnitine shuttle is a complex consisting of three enzymes whose function is to transport the long-chain fatty acids into the mitochondria. Here, its pharmacological modification was used to test the hypothesis that shifting metabolism to lipid oxidation exacerbates the HD symptoms. Behavioural and transcriptional analyses were carried out on HD Drosophila model, to evaluate the involvement of the carnitine cycle in this pathogenesis. Pharmacological inhibition of *CPT1*, the rate-limiting enzyme of the carnitine cycle, ameliorates the HD symptoms in Drosophila, likely acting on the expression of carnitine-related genes.

## 1. Introduction

Huntington’s disease (HD) is a neurodegenerative disorder due to the abnormal expansion of the unstable CAG triplet repeats in exon 1 of the *huntingtin* (*Htt*) gene and the resulting formation of an abnormal polyglutamine tract in the protein [1]. The mutated misfolded HTT protein (mHTT), featured by a polyglutamine tract over 35 repetitions long (polyQ), is prone to the formation of aggregates that can lead to a global collapse in neuronal proteostasis [2,3], defective energy metabolism, and oxidative stress. Further, mHTT plays a key role in reprogramming of the histone modification H3K27me3 through activation of the related methyltransferase polycomb repressive complex 2 (PRC2) [4]. Human HUNTINGTIN protein (HTT) is extensively expressed in the brain and testis and is localised in both the cytoplasm and nucleus. It is involved in several cellular processes that are drastically altered in HD [5,6]. The most important pathological feature of HD is the selective degeneration of the striatal and cortical neurons that project from the cortex to the striatum, leading to cell death [7]. For this reason, HD patients present with a plethora of neurological symptoms that manifest in a deterioration of motor, cognitive, and psychiatric functions [8]. Recent findings shed light on other pathological features as potential targets for therapeutic intervention [9], such as mitochondrial dysfunction and metabolic alterations [5,10,11], which are responsible for energy deficits through multi-factorial and integrated mutant HTT (mHTT)-induced mechanisms [12]. Since both HD patients and models exhibit mitochondrial abnormalities with defective respiratory chains, increased oxidative stress and ATP deficit [13], the bioenergetic alterations, such as increased fatty acids (FAs) metabolism versus glucose oxidation are thought to represent a compensatory response to impaired glucose-based energy production [14,15,16]. In a Drosophila model of HD, pharmacological inhibition of the carnitine system was reported to have an ameliorative effect on lifespan and locomotor dysfunction, two major features of the HD disease [11]. These data are consistent with the hypothesis that inhibition of the carnitine system in these HD flies favours a decrease in fatty acid oxidation and a parallel increase in glucose oxidation.

Teglicar ((R)-*N*-(tetradecylcarbamoyl)-aminocarnitine), also known as ST1326, is a reversible inhibitor of the 1A isoform of carnitine palmitoyl transferase (*CPT1A*), the rate-limiting enzyme of the carnitine shuttle (CS), a mitochondrial machinery consisting of three proteins: two carnitine palmitoyl transferases, *CPT1* and *CPT2*, and the carnitine acylcarnitine translocase (*CACT*), whose function is to transfer the activated long-chain FAs in the reverse direction into the mitochondrial matrix for β-oxidation [17,18]; to complete this cycle, carnitine acetyltransferase (*CrAT*), localised in the matrix, converts the acetyl-CoAs produced into acetylcarnitines, allowing their export to the cytosol. Long-chain FAs cannot pass through mitochondrial membranes, so *CPT1* conjugates them with carnitine molecules to produce acylcarnitines; in this form, the long-chain FAs can be transported to the mitochondrion via the CS [19]. *CACT* allows the acylcarnitines to cross the inner mitochondrial membrane by exchanging them with free carnitine/acetylcarnitine molecules from the mitochondrial matrix and *CPT2* conjugates the long-chain acylcarnitines back to coenzyme A after translocation to the mitochondrion [20]. By inhibiting *CPT1*, Teglicar impairs the oxidation of FAs and the availability of cytosolic acetyl-coenzyme A (acetyl-CoA) [21]. It has been tested in haematopoietic [22] and bladder tumours [23], where it targets metabolic dysregulations critical for cancer cell survival [21], and in diabetes, where it reduces gluconeogenesis and improves glucose homeostasis [24]. 

As described, impaired mitochondrial activity and altered energy metabolism are clearly associated with HD, and several lines of research have focused on the involvement of carnitine and lipid metabolism in the disease [25,26] and how their modulation may help to improve symptoms [27]. 

We decided to investigate the effect of reversible *CPT1* inhibition on a transgenic model of HD by administering Teglicar to *Drosophila melanogaster*. 

Drosophila is an attractive in vivo model for conducting functional studies of human genes and their disease variants because of its short life cycle, easily manipulable genome, similarity to human genes and organs, complex behaviour and extensive genetic toolkit. Finally, the fly is subject to few legal restrictions and avoids the ethical problems that exist with other higher animals for in vivo studies. In recent decades, the fly has been used extensively for modelling neurodegenerative diseases [28,29], including HD. The ease of creating transgenic flies allowed to develop Drosophila models expressing the mutated protein encoded by the first exon of human Htt or by its full-length cDNA with an extended polyglutamine (polyQ) stretch near the *N*-terminus (hFLHD-128Q). In our experiments, we used a Drosophila strain containing the human full-length HTT with a polyQ stretch of 128 repeats, which represents a severe form of the disease. To allow temporal and spatial expression by the GAL4-UAS system, the flies carry an upstream activating sequence (UAS) upstream of the human hFLHD-128Q gene. The binary GAL4-UAS system is one of the most effective tools available in Drosophila to modulate gene expression with spatial and temporal specificity. By expressing hFLHD-128Q in the nervous system with the pan-neuronal elav-GAL4, flies recapitulated the major features of HD neuronal dysfunction, such as decreased lifespan, progressive accumulation of aggregates in the cytoplasm, mitochondrial dysfunction, progressive loss of coordination and age-related motor impairment [14]. In addition, these hFLHD-128Q transgenic flies also exhibited disruption of lipid levels, with defective cellular lipid accumulation [30] and consequent energy deficiency [31].

The carnitine transport system of Drosophila is essentially similar to that of humans [18]. The *CPT1* protein, encoded by a single gene called withered (*whd*), shows greater similarity to human isoform A; *whd* mutants show increased lipid storage and are highly sensitive to starvation and oxidative stress [32]. The *CPT2* protein has 67% amino acid sequence similarity to the human protein, and its deficiency leads to an interesting accumulation of triacylglyceride-filled lipid droplets in the brain [33]. Finally, the protein that functions as a carnitine-acylcarnitine transporter, since it was able to rescue a yeast *CACT* deletion strain, is encoded by the homologous *CACT* gene called congested-like trachea (*colt*) [34]. Recently, it has been suggested that the Drosophila gene *CG6356* is the putative orthologue of the human SLC22A16 gene, which encodes a high-affinity carnitine transporter and thus plays a critical role in the cellular uptake of carnitine [18,35]. In addition, genes involved in the β-oxidation of FAs have been shown to play a role in age-related neurodegeneration in Drosophila [33,36]. These include *CG10814* gene, the putative orthologue of the human *γBBD* gene [18,36], which catalyses the final step of carnitine biosynthesis, and *dHNF4*, which is thought to be the master regulator of β-oxidation in the fly [37,38].

In this contest, our first aim was to test the hypothesis that the dietary administration of Teglicar would alleviate motor dysfunction and prolong the survival of a transgenic HD Drosophila model. At same time we reasoned that dietary administration of coconut oil, a mixture of medium and short-chain fatty acids that freely enter the mitochondria to provide lipids for β-oxidation, would enhance HD symptoms. Finally, to unravel potential mechanisms by which Teglicar treatment may have biological effects, we analysed the gene expression of several genes involved in the carnitine cycle in this pathogenesis.

For the first time, we demonstrate that Teglicar can ameliorate some mitochondrial dysfunction in a Drosophila HD model and that these beneficial effects are correlated to a restore of the carnitine-related genes; at the same time, we reported that the availability of lipids for β-oxidation plays an important role in the development of HD symptoms.

## 2. Results

### 2.1. Pharmacological Inhibition of CPT1 Alleviates Locomotor Defects in the Huntington’s Drosophila Model

**Figure 1:**
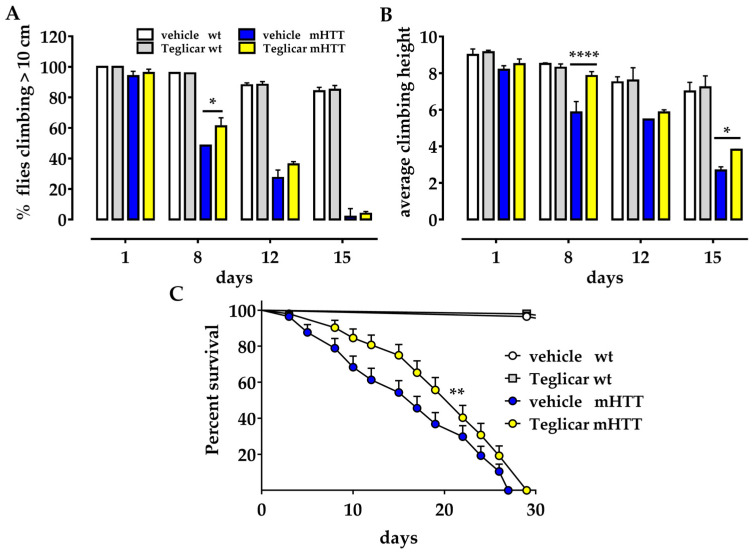
Teglicar suppresses the neurodegenerative phenotype in a Drosophila Huntington model. (**A**) Climbing assay. The percentage of Huntington’s flies (mHTT) that received chronic treatment with Teglicar climbs better than untreated Huntington’s age-matched siblings (vehicle) and this difference was significant 8 days after eclosion. There are no significant differences in the percentage of climbing flies 1, 12, and 15 days after eclosion. (**B**) Average height. Huntington’s flies chronically treated with Teglicar climb significantly higher than the untreated control at 8 and 15 days after eclosion. (**C**) Lifespan assay. After chronic feeding with Teglicar, mHTT flies exhibited significantly higher survival rate in comparison to untreated mHTT flies. Maximum survival is significantly increased in Teglicar-treated flies compared to untreated flies (29 vs. 27); further, 50% of Teglicar-treated flies are still alive around day 20, while 50% of untreated control flies are alive around day 15 (median survival: 22 vs. 17 days). In all these assays, Teglicar wt flies did not show any differences in comparison to wt untreated flies in the time period examined. Values represent means ± SEM (*n* = 3), *n* = 60/cohort. For A and B, student’s *t* test was used to calculate significance. Survival curves were analysed using Mantel-Cox analysis (*n* = 2). * *p* < 0.05; ** *p* < 0.01; **** *p* < 0.0001.

To investigate the effect of reversible inhibition of *CPT1*, we used a well-established Drosophila model for Huntington’s disease [11] (Q128HD-FL) in which the human HTT complementary DNA (cDNA) with 128 glutamine repeats is expressed in all neuronal tissues (genotype: elav-Gal4/+; UAS-hFLHD-128Q/+;). These flies experience a very aggressive course of HD disease, showing a survival of ~80% by day 6, ~50% by day 14, ~20% by day 22, and ~5% by day 27, as compared to 100% of the parental strain UAS-hFLHD-128Q used as a wild-type control. Due to a neuronal impairment, Huntington’s flies also exhibit a dramatic defect in locomotion that can be measured by the climbing assay, which exploits the strong negative geotaxis behaviour of Drosophila. The climbing ability of Q128HD-FL flies was 100% at day 1, 80% by day 3, 50% by day 8, 30% by day 9, and lost by day 15 in comparison to the wt control. We treated the HD fly model with the reversible *CPT1* inhibitor Teglicar (50 μM) throughout adulthood, from fly eclosion to death. We tested Huntington’s flies on the day of eclosion (day 1) and 8, 12, and 15 days post eclosion. We calculated the percentage of flies that could climb over 10 cm and found that treatment with Teglicar significantly improved the climbing ability of flies 8 days after eclosion compared to untreated age-matched sibling flies (61.1% ± 5.5 versus 48.4% ± 0.1 * *p* < 0.05) (Figure 1A) and delayed the onset of symptoms. Although there are no differences between treated and untreated flies on the last days of treatment (12 and 15 days after eclosion), the average climbing height reached by treated and untreated flies on the day of eclosion and 8, 12, and 15 days after clearly show that flies treated with Teglicar climb higher than untreated sibling flies. As shown in Figure 1B, the average climbing height of Teglicar-treated flies compared to untreated flies at 8 and 15 days was 7.8 cm ± 0.2 versus 5.8 cm ± 0.6; **** *p* < 0.0001; 3.8 cm ± 0.3 versus 2.7 ± 0.03; * *p* < 0.05, respectively (Figure 1B).

### 2.2. Pharmacological Inhibition of CPT1 Increases Survival in the Huntington’s Drosophila Model

Because Huntington’s flies exhibit age-dependent phenotypes, we measured their lifespan and survival rate to determine whether treatment with Teglicar could slow down the disease. Huntington’s flies treated with Teglicar have a maximum lifespan of 29 days compared to 27 days in untreated sibling flies; furthermore, their curves differ significantly (** p < 0.001) (Figure 1C). A comparison of the survival curves of the treated and untreated flies shows that they overlap in the first 3 days and diverge thereafter due to the better survival rate of the flies treated with Teglicar (median survival 22 versus 17). In fact, the curve of the treated flies drops on day 15 compared to the curve of the untreated flies, which drops on day 5. These data suggest that Teglicar can slow down the progression of the disease. 

### 2.3. Dietary Supplementation with Medium-Chain Fatty Acids Affects the Locomotion of Huntington’s Larvae

**Figure 2:**
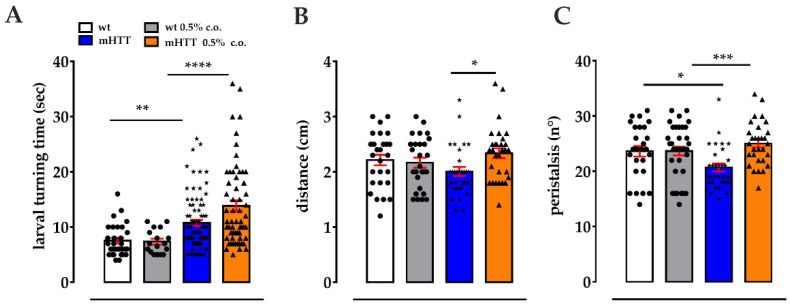
Coconut oil supplementation exacerbates the neurodegenerative phenotype in 3rd instar mHTT larvae. (**A**) Larval turning. mHTT 3rd instar larvae fed on a 0.5% coconut oil supplemented diet (mHTT c.o.) turn to the ventral side significantly more slowly than Q128HD-FL larvae fed on normal food (mHTT), which in turn already turn to the ventral side more slowly than wild-type control. (**B**) Distance. The mHTT larvae fed on food supplemented with 0.5% c.o. travel a significantly greater distance in 30 s than the mHTT larvae fed on normal diet. (**C**) Peristalsis. Number of peristalsis in 30 s during crawling significantly increases in mHTT 0.5% c.o. larvae in comparison to untreated mHTT larvae. In all these assays, no differences were revealed between treated and untreated wt larvae. Values represent mean ± SEM (*n* = 3). Sidak’s one-way ANOVA pairwise multiple comparisons test was used to calculate significance. * *p* < 0.05; ** *p* < 0.01; *** *p* < 0.001; **** *p* < 0.0001. •: values of individual wt larvae; ★, ▲: values of individual mHTT c.o.-treated and untreated larvae, respectively.

Due to low glucose levels in the brains, HD patients and models show a switch from glycolysis to FAO. This compensatory response to impaired glucose-based energy maintains proper ATP levels but at the same time lead to an increase flux in ROS and finally to a loss of neurons in a specific brain area [11,12,13]. The CS ensures that long- and very long-chain FAs enter the mitochondrion for β-oxidation [17,18,39], while the medium- and short-chain FAs can pass through the mitochondrial membranes [40]. Teglicar is known to inhibit the activity of *CPT1*, the rate-limiting enzyme of CS, and to restrict the import of long-FAs into the mitochondrion. Based on the idea that the administration of Teglicar to HD flies significantly improved neurodegenerative damage in these flies through a decrease in fatty acid oxidation, we wondered whether the availability of medium-chain fatty acids for β-oxidation might play an important role in the development of HD symptoms. To assess the effect of these lipids on neurodegeneration, we added coconut oil containing a mixture of medium-chain fatty acids to the fly diet and carried out several analyses on mHTT larvae and adult females. mHTT 3rd instar larvae exhibit abnormal movements in comparison to wt control that can be quantified by several tests. We tested both wt and mHTT 3rd instar larvae fed cornmeal-medium enriched with 0.5% coconut oil for self-righting behaviour. This involved examining how quickly the larva turns back onto its ventral side when lying on its dorsal side [41,42]. This movement results from the coordination of successive contractions of body segments, and since abnormal responses to turning over have been observed in a fly model of amyotrophic lateral sclerosis [43], it can be considered a functional indicator of the neurodegeneration underlying the change in locomotion. Notably, wt larvae fed 0.5% coconut oil did not show any differences with respect to untreated age-matched larvae; instead, mHTT larvae fed 0.5% coconut oil turned to the ventral side significantly slower than mHTT larvae fed normal food (13.8 s ± 0.9 versus 10.7 s ± 0.5; **** *p* < 0.0001), which in turn are significantly slower than wild-type larvae (10.7 s ± 0.5 versus 7.53 s ± 0.5; ** *p <* 0.001) (Figure 2A). We then performed the crawling assay, evaluating the distance travelled and the number of peristaltic waves in 30 s. The mHTT larvae fed with 0.5% coconut oil covered a significantly longer distance (2.4 cm ± 0.09 versus 2.00 cm ± 0.08; * *p* < 0.05) compared to the mHTT larvae fed a normal food (Figure 2B); instead, no differences were revealed between treated and untreated wt larvae. Referring to the number of peristaltic waves, mHTT larvae fed 0.5% coconut oil showed an increase compared to mHTT larvae fed a normal diet (26 ± 0.7 versus 20.7 ± 0.7; *** *p* < 0.001) (Figure 2C). The mHTT larvae fed a normal diet showed a lower number of peristaltic waves compared to the wt larvae fed a normal food, which in turn resulted in being unchanged following coconut oil treatment (Figure 2C).

### 2.4. Dietary Supplementation with Medium-Chain Fatty Acids Worsens the Climbing Ability of mHTT Flies

**Figure 3:**
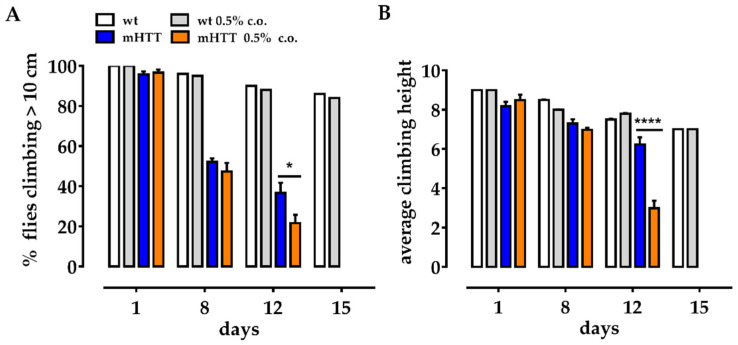
Coconut oil supplementation exacerbates the neurodegenerative phenotype in adult mHTT flies. (**A**) Climbing assay. The percentage of mHTT flies fed a normal diet (mHTT) climbed better than the mHTT flies fed coconut oil (mHTT 0.5% c.o.) 8 and 12 days after eclosion. (**B**) Average height. mHTT flies fed a normal diet reach an average climbing height significantly higher than mHTT 0.5% c.o. 12 days after eclosion in comparison to mHTT age-matched untreated sibling flies. Values represent mean ± SEM (*n* = 3). Student’s *t* test was used to calculate significance. * *p* < 0.05; **** *p* < 0.0001.

To address motor function of adult flies after dietary supplementation with medium-chain FAs, we measured the climbing ability of mHTT flies fed 0.5% coconut oil (since the larval stage) at days 1, 8, 12, and 15 after eclosion. Our data show that feeding coconut oil from the beginning of larval life worsens the motor ability of mHTT flies compared to age-matched mHTT flies fed a normal food. As shown in Figure 3, at days 8 and 12 post eclosion, both the percentage of mHTT flies climbing over 10 cm and the average climbing height reached were significantly lower in mHTT flies fed 0.5% coconut oil (Figure 3B).

### 2.5. Transcriptional Profiles of Several Genes Involved in the Drosophila L-Carnitine Cycle Are Deregulated in Q128HD-FL Flies

**Figure 4:**
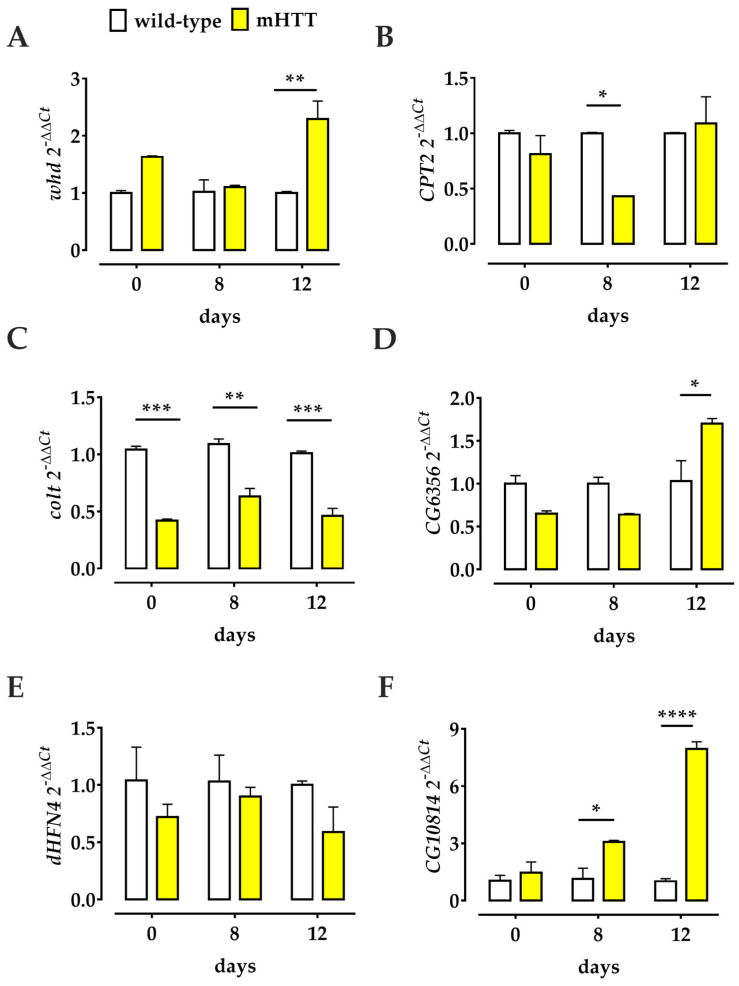
Altered transcriptional profiles in the heads of Q128HD-FL flies. Comparison of the relative gene expression of *whd* (**A**), *CPT2* (**B**), *colt* (**C**), *CG6356* (**D**), *dHFN4* (**E**), and *CG10814* (**F**) genes in 1-, 8-, 12-days old mHTT and age-matched wt flies. All CS component genes are affected by the HTT mutation: *whd* is overexpressed, while *CPT2* and *colt* are both downregulated in the heads of Q128HD-FL flies compared to the wt. The hypothetical carnitine transporter *CG6356* is overexpressed in the Huntington model in comparison to the wt. The master regulator of lipid oxidation *dHNF4* remains unchanged at each time point examined. *CG10814*, which encodes a carnitine biosynthetic enzyme was gradually over-expressed as the disease progresses in comparison to healthy wt flies. Relative gene expression was calculated with respect to the *rp49* gene and control samples. (q)RT-PCR was performed on three different experiments, and the results are expressed as the mean of the values obtained (mean ± SEM). Sidak’s one-way ANOVA pairwise multiple comparisons test was used to calculate significance. * *p* < 0.05; ** *p* < 0.01; *** *p* < 0.001; **** *p* < 0.0001.

To further evaluate the role of Teglicar in our fly HD model, we first wondered what happens to the transcriptional regulation of several genes involved in the Drosophila L-carnitine cycle in the presence of the mHTT protein. To this end, we performed a (q)RT-PCR analysis on RNA extracted from the heads of mHTT adult flies and wt flies as controls. To analyse the CS components in the Huntington’s Drosophila model, we used specific primers for the *whd*, *CPT2*, and *colt* transcripts; the *whd* and *CPT2* genes are orthologues of the human *CPT1* and *CPT2* genes, respectively, while *colt* gene is the orthologue of the human *CACT* gene [19]. We compared the expression of these genes in heads of mHTT flies at the day of eclosion (day 1), at days 8 and 12 after eclosion with the age-matched parental control used as the wt strain. Our results showed significant deregulation of CS gene expression in the head of Q128HD-FL flies: *whd* was significantly overexpressed 12 days after eclosion, while *CPT2* was significantly downregulated 8 days after eclosion, as was *colt*, which was significantly downregulated on the day of eclosion and 8 and 12 days after (Figure 4). These data suggest that in Q128HD-FL flies, transcriptional deregulation of all genes encoding the protein involved in carnitine shuttle may play a pathogenic role. In addition, we also examined the expression of several other genes involved in the carnitine cycle in Drosophila: the *CG6356* gene, the putative orthologue of the human *SLC22A16* gene [18] that encodes the fly’s hypothetical carnitine transporter; the *dHNF4* gene, the master regulator of β-oxidation in the fly [37]; and the *CG10814* gene, a putative orthologue of the human *γBBD* gene, that encodes the enzyme that catalyses the final step of carnitine biosynthesis [36]. Our data clearly indicated transcriptional deregulation of the *CG6356* and *CG10814* genes at all days examined; instead, the expression of *dHNF4* remained unchanged at all time points examined. The level of *CG6356* was significantly higher 12 days after eclosion in Q128HD-FL flies than in the wild-type control. Interestingly, the level of *CG10814* transcript gradually and significantly increased 8 and 12 days after eclosion (Figure 4). 

### 2.6. Pharmacological Inhibition of CPT1 Activity Affects Both Component Genes of the CS and the Transcriptional Profiles of CG6356 and CG10814

**Figure 5:**
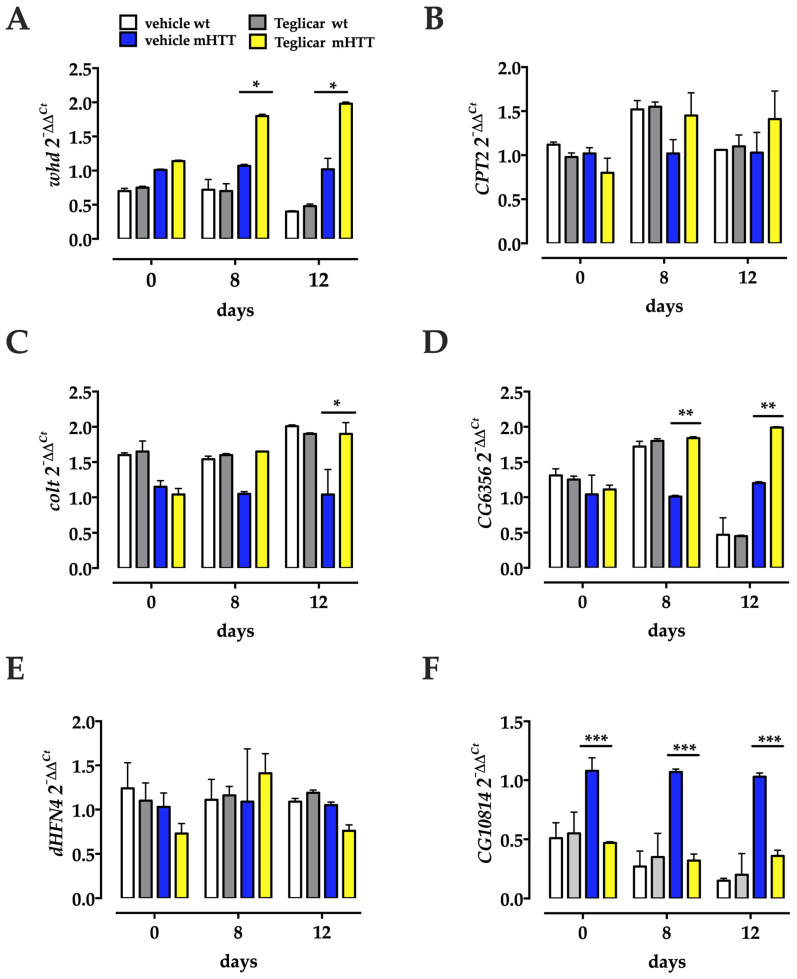
Heads transcriptional profiles of Drosophila carnitine cycle genes are altered by pharmacological inhibition of *CPT1* activity. Two of the CS component genes are significantly overexpressed after Teglicar treatment at 8 and 12 days post-eclosion, *whd* (**A**) and *colt* (**C**), compared to the untreated control (vehicle mHTT). *CG6356* (**D**) is upregulated compared to untreated controls (vehicle mHTT) at both 8 and 12 days post-eclosion, while *CG10814* (**F**) is downregulated compared to untreated controls (vehicle mHTT) at days 1, 8, and 12 post-eclosion. *CPT2* (**B**) and *dHNF4* (**E**) remain unchanged at each time point examined. Relative gene expression was calculated with respect to the *rp49* gene and control samples. Notably, for each gene, no transcriptional alterations were revealed between Teglicar-treated and vehicle-treated wt flies. (q)RT-PCR was performed on three different experiments, and the results are expressed as the mean of the values obtained (mean ± SEM). Sidak’s one-way ANOVA pairwise multiple comparisons test was used to calculate significance. * *p* < 0.05; ** *p* < 0.01; *** *p* < 0.001.

After determining the transcriptional regulation of carnitine cycle genes in the HD flies, we asked whether oral administration of the reversible *CPT1* inhibitor Teglicar could have any effect on the transcription of these genes in the brain. We performed a (q)RT-PCR on RNA extracted from the heads of Huntington’s flies chronically treated with Teglicar from their eclosion to day 12 post eclosion and from the heads of untreated, age-matched Huntington’s flies. We found that in HD flies fed Teglicar, the level of two of the transcripts (*whd* and *colt*) involved in CS increased with the progression of pharmacological treatment (Figure 5), with a significant increase in late symptomatic flies, 12 days post eclosion, compared to age-matched untreated flies (Figure 5). Next, we performed this analysis for the other genes involved in the carnitine cycle that were previously studied. Our results show that, like the CS genes, *CG6356* was significantly upregulated at both days 8 and 12 post-eclosion, compared to age-matched untreated flies. *dHF4* remained unchanged at all time points. In contrast, *CG10814* resulted in being downregulated in comparison to age-matched untreated flies at 1, 8, and 12 days post-eclosion (Figure 5). Notably, none of the examined gene showed significantly altered expression level in age-matched Teglicar treated and untreated wt flies.

### 2.7. The Genes for the CS Components Are Upregulated by Dietary Supplementation with Medium-Chain Fatty Acids, as Are CG6356, dHNF4 and CG10814

**Figure 6:**
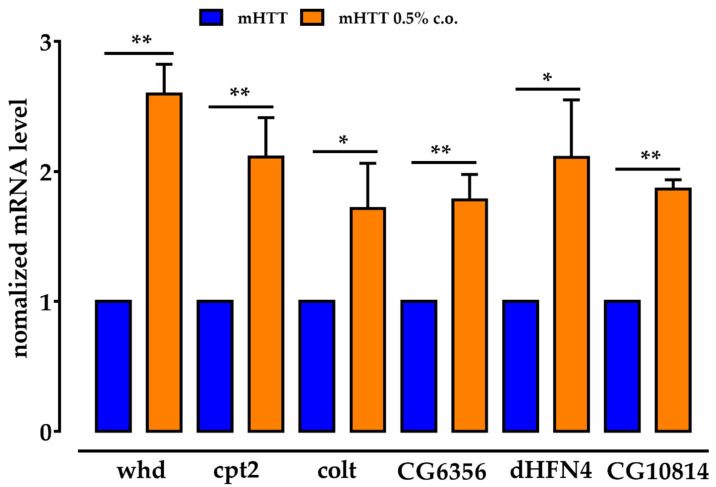
Dietary supplementation with coconut oil stimulates the expression of carnitine cycle genes in Drosophila. Administration of 0.5% coconut oil significantly overexpressed the key genes encoding for the components of the CS (*whd*; *CPT2* and *colt*) compared to untreated mHTT, as well as *CG6356*, *dHNF4,* and *CG10814.* None of the examined gene showed significantly altered expression level in age-matched c.o. treated and untreated wt flies. Relative gene expression was calculated with respect to the *rp49* gene and control samples. (q)RT-PCR was performed on three different experiments, and the results are expressed as the mean of the values obtained (mean ± SEM). Sidak’s one-way ANOVA pairwise multiple comparisons test was used to calculate significance. * *p* < 0.01; ** *p* < 0.001.

Considering the close correlation between lipid β-oxidation and CS, to assess the effects of dietary supplementation with medium-chain FAs, we analysed the expression of *whd*, *CPT2*, *colt*, *CG6356*, *dHNF4*, and *CG10814* in Q128HD-FL larvae fed a 0.5% coconut oil-supplemented diet. All analysed genes were significantly overexpressed compared to larvae fed with normal diet, allowing us to confirm that lipid excess affects the components of CS and genes involved in carnitine transport, lipid oxidation, and carnitine biosynthesis (Figure 6). Notably, dietary 0.5% coconut oil did not affect the transcriptional expression of all the examined genes in the heads of the wt control adult females.

## 3. Discussions

In this paper, we evaluated the effects in vivo of the reversible carnitine palmitoyltransferase 1 inhibitor Teglicar on a HD Drosophila model. Our results showed that dietary Teglicar treatment significantly ameliorated the status of HD flies, by acting on the expression of carnitine-related genes.

It is known that in the HD brain, glycolytic striatal cells undergo a metabolic switch from glycolysis to FAO. This metabolic change maintains proper ATP levels, but at the same time it leads to an increase in superoxide radical anion production, causing mitochondrial dysfunction and neuronal cell death. Researchers report that blocking mitochondria reactive oxygen species (ROS) with an antioxidant compound (XJB-5-131), or decreasing FAO by reducing cellular carnitine levels via THP (Meldonium), HD symptoms were mitigated [44]. In addition, in a HD Drosophila model, an ameliorative effect of HD symptoms was reported following metabolic reprogramming to glucose, by overexpression of Glucose 6 Phosfate Dehydrogenase [45] and a glucose transporter [46].

Teglicar is a reversible inhibitor of the 1A isoform of carnitine palmitoyl transferase (CPT1A), the rate-limiting enzyme for fatty acid import in mitochondria that impairs the oxidation of FAs and the availability of cytosolic acetyl-coenzyme A (acetyl-CoA) [21]. This reduction of reliance of the cell on FAO leads to a metabolic shift toward glucose oxidation. 

To test the hypothesis that blocking the translocation of long Fas into mitochondria via *CPT1* could enhance glucose utilization and ameliorate Huntington’s symptoms [10,26,30,47], we treated HD flies with Teglicar [24]. At the same time, based on the idea that medium and short-chain- fatty acids did not depend on the CS to cross mitochondrial membranes, we reasoned that these FAs could freely enter the mitochondria and enhance oxidative stress and HD symptoms. So, we treated HD flies with coconut oil, a mixture of medium-chain FAs that cross mitochondrial membranes without involvement of the CS to provide lipids for β-oxidation [48].

We decided to administer Teglicar to the adult flies to evaluate its potential use as a drug to alleviate the symptoms of an early symptomatic organism, and to administer the coconut oil to both larvae and adult flies to test whether it can induce neurodegeneration at a very early stage of development. The flies were given chronic treatment with Teglicar and tested for negative geotaxis ability and survival, showing improvement in HD symptoms. The HD flies showed severe locomotion impairment and have a short life [49,50]. According to our data, treatment with Teglicar significantly improves locomotion and delays the onset of the disease. In the climbing test, treatment with Teglicar increased the percentage of flies climbing on the empty vials on day 8 (symptomatic stage) but not on days 12 and 15 after eclosion (late symptomatic stages), although the flies climbing on the vials reached a significantly higher mean height compared to the untreated controls, indicating a delay in the exacerbation of symptoms. In contrast, when we tested HD coconut oil-fed 3rd instar larvae and adult flies to assess their locomotion, we found a worsening of HD symptoms. 

Our data showed that coconut oil-fed larvae turned back to their ventral side more slowly than control flies, but they covered a significantly greater distance with a higher number of peristaltic waves, most likely as a result of the larvae’s abnormal behaviour during crawling, such as frequent stops and “head swings“ [51]. Like the larvae, the adult flies that performed the climbing experiment also showed impaired locomotion. Taken together, these data suggest that disease progression is influenced by a modulation of lipid β-oxidation, involving CS in the translocation of FAs to mitochondria. Since *CPT1* plays a key role in mitochondrial processes, many studies have targeted its pharmacological or genetic inhibition [52,53,54] to show that the shift from glucose to lipid metabolism is crucial for disease development. Metabolic deregulation has been proposed as a crucial point in the pathogenesis of several neurodegenerative diseases, such as ALS [55], myotonic dystrophy [56], Alzheimer’s, and Parkinson’s diseases [57], where the balance between glucose and lipid oxidation is disturbed, as is the case in HD. For HD, enhanced lipid β-oxidation leads to an increase in radical oxygen species and oxidative stress, resulting in defective respiratory chains and ATP deficit [39], which further drives metabolic deregulation and mitochondrial damage on the one hand, and triggers neurodegeneration on the other [49,52]. Many CPTs and carnitine transporter inhibitors have been tested to target metabolic deregulation in neurological diseases [14,51]. Here we used Teglicar for the first time in HD flies, taking advantage of its ability to reversibly and selectively inhibit *CPT1*, making it suitable for a potential therapeutic approach. 

Before examining the effects of administering Teglicar and coconut oil, we found that HD caused changes in the expression of both the components of CS and other genes involved in the carnitine cycle that we examined. Our results clearly indicate that the *whd* gene is upregulated while *CPT2* and *colt* genes are downregulated compared to wild-type, similar to what was shown in a study using an ALS Drosophila model [48]. This could be explained as an attempt to compensate for a severely impaired metabolic state typical of neurodegenerative diseases [58], in which the cell allows fuel to be imported into the mitochondrion. Moreover, this could be considered as an indication of the role of CS in neurodegeneration. We then performed the same analysis after administration of Teglicar and coconut oil. This showed that both affected the components of CS and upregulated their expression. The Teglicar-induced *whd* gene overexpression appears to be the result of a transcriptional compensation mechanism caused by the inhibition of protein activity due to Teglicar, while the overexpression of *colt* gene may indicate that, given the inhibition of the rate-limiting enzyme of CS, there is an accumulation of its substrate in the cytosol that induces transcriptional regulation to increase its mRNA levels [59,60]. The CS components are also upregulated by dietary supplementation with coconut oil: just as with Teglicar administration, there is an accumulation of the lipid substrate, albeit for different reasons, because coconut oil is a mixture of short- and medium-chain FAs that cross mitochondrial membranes, but a small percentage consists of long-chain FAs that use the CS to be translocated to the mitochondrion [61]. We hypothesise that the cytoplasmic accumulation of lipids may cause the cell to increase the mRNA levels of the CS components to get rid of the excess of long-chain FAs. In view of these results, the observed neurodegenerative phenotype in Drosophila, its improvement by Teglicar, and its deterioration by coconut oil, does not seem to be related only to the expression of the CS components. 

We therefore also examined the transcriptional profiles of the following genes under the above conditions: the *CG6356* gene encoding the high-affinity carnitine transporter, a putative orthologue for human SLC22A16 [18], the *dHNF4* gene, the master regulator of β-oxidation in the fly [37], and the *CG10814* gene, encoding for the enzyme that catalyses the final step of the carnitine biosynthesis pathway—a putative orthologue for human *γBBD* [18,36]. In the brains of HD flies, *CG6356* mRNA levels are upregulated, while *dHNF4* remains unchanged and *CG10814* is significantly overexpressed with disease progression. This deregulation of mRNA levels may be the result of metabolic dysfunction of fatty acid β-oxidation in the nervous tissue of HD flies, which is related to the impaired mitochondrial activity previously reported in this HD Drosophila model [14]. 

Of particular interest was the overexpression of the *CG10814* gene. It has been reported that this gene is upregulated in the Drosophila brain with age and that its genetic downregulation abrogates the functional decline of the brain with age [36]. Consistent with these data, our analysis clearly shows that after chronic treatment with Teglicar, the expression of this gene was downregulated compared to untreated flies. *CG6356* gene is also modulated by treatment with Teglicar compared to untreated flies. Following dietary supplementation with coconut oil, which significantly worsens disease progression in HD flies as shown by behavioural analysis, *CG6356*, *dHNF4*, and *CG10814* genes show upregulation, supporting the idea that fatty acid β-oxidation in the brain of the HD fly may play an important role in the neuronal decline typical of the HD disease. The observed upregulation of *CG6356*, *dHNF4*, and *CG10814* genes might be due to the accumulation of lipid substrate in the cytosol [62], as their proteins are involved in carnitine transport [18,35] and lipid oxidation [37], respectively. As for *CG10814*, we can summarise that it is upregulated in the HD brains and after coconut oil administration when the Drosophila phenotype is impaired, and downregulated by Teglicar treatment when symptoms of neurodegeneration improve. We hypothesise that alteration of the *CG10814* gene is responsible for the neurodegenerative phenotype in HD Drosophila: the gene encoding the enzyme involved in the final step of carnitine biosynthesis is likely to be downregulated when the cell does not require high carnitine concentrations, as its activity is modulated by CS, as was the case with Teglicar treatment. Conversely, its overexpression indicates abnormal mitochondrial activity that shifts to lipid metabolism, which requires carnitine for translocation of FAs into the mitochondrial matrix. *CG10814* gene, which encodes a protein involved in the carnitine synthesis, also plays a key role in the translocation of FAs through the CS [18], so we can speculate that its overexpression also plays a role in lipid β-oxidation. The mechanism underlying these changes in expression remains to be deciphered, but these results seem to suggest that modulation of lipid β-oxidation by carnitine shuttling is involved in HD pathogenesis [31].

In conclusion, the link between Huntington’s neurodegeneration and metabolic effects is still not clear. However, therapeutic strategies that use small compounds to influence mitochondrial energy processes may help in the development of appropriate treatments to slow the progression of the disease.

## 4. Materials and Methods

### 4.1. Drosophila Stocks and Crosses

Flies were reared on standard cornmeal-agar with a 12-h on–off light cycle at 25 °C. The fly stocks used in this study were obtained from the Bloomington Stock Center (Bloomington, IN, USA): P{UAS-HTT.128Q.FL}f27b-8765 w; P{GAL4-elav.L}2-1521 w [*]. The expression of huntingtin-containing polyglutamine was driven by the bipartite expression system upstream activator sequence (UAS)-GAL4 in transgenic flies [63]. To obtain the strain carrying the Huntington’s mutation under the neuronal promoter, we crossed the females with the pan-neural driver elav-Gal4 to males from the UAS HTT128QFL strain. In all assays, the parental strain UAS HTT128QFL was used as a wild-type control. For all tests performed on adult organisms, we used only female flies. 

### 4.2. Teglicar Treatment

Teglicar (Sigma-Aldrich; Avanti Polar Lipids 870853P; Merk Life Science S.r.l., 20149 Milano, Italy) was added to the surface of the assay fly food (AF, 2% agar, 10% powdered yeast, 10% sucrose, 0.1% Nipagin) at the final concentration of 50 μM, and dried with gentle agitation for 3 h at room temperature [64]. This food was used for rearing the experimental flies, while the controls were reared in AF without Teglicar. Water was added in equal amounts for all the feeding conditions. The criteria of the selected concentration were based on our preliminary findings. To check if the presence of the Teglicar in the assay fly food could affect the feeding of HD and parental flies, the red food dye no. 40 was added to AF medium supplemented with the molecule used [11]. The flies were allowed to feed on the dye-supplemented medium for one day, and their abdominal colouring was examined under a stereomicroscope.

### 4.3. Climbing Assay

Climbing assays were performed on female flies on the day of eclosion (day 1) and 8, 12, and 15 days after eclosion, corresponding to the presymptomatic, symptomatic, and late symptomatic stages of the disease, respectively. Flies were collected, briefly anesthetized with CO2, and sorted by sex. Then, cohorts of no more than 20 flies were placed in vials containing AF supplemented with or without Teglicar, and reared at 28 °C. On the days of the experiment, they were transferred to graduated empty plastic vials (18 × 2.5 cm) and acclimated for 30 min. They were then gently tapped on the bottom of the vial, and the number of flies that climbed above the 10-cm mark within 30 s after the tap was recorded. Each group was tested twice, with a 1-min rest period between each trial. The number of flies per group that reached the 10-cm mark was calculated as a percentage of the total number of flies, and the average height for each experimental group was also measured. For each condition, 3 groups of 20 flies each were tested in the marked tube in 3 independent experiments, and the data were expressed as an average of the replicates.

### 4.4. Lifespan Analysis

Newly hatched adult flies were collected, briefly anesthetized with CO_2_, and sorted by sex. Then, cohorts of no more than 20 flies were placed in vials containing AF, supplemented with or without Teglicar, and reared at 28 °C. They were transferred to new vials with fresh food every two days, and deaths were recorded at each transfer. The experiment ended when there were no more live flies in the vials. The lifespan was measured in two independent experiments (total *n* = 100) per treatment.

### 4.5. Medium-Chain FAs Fly Food Supplementation

A total of 0.5% virgin coconut oil was added to the standard fly food made of cornmeal and agar, then the food was dispensed into vials and cooled. This food was used for rearing the experimental 3rd instar Huntington’s larvae. The 3rd instar Huntington’s larvae used as controls were reared in fly food without coconut oil.

### 4.6. Larval Turning 

The experiment was performed as previously described [39]. Briefly, 3rd instar larvae were collected from both the standard fly food supplement with coconut oil, washed three times in distilled water, and placed on a 3% agar plate at room temperature. After a few minutes of acclimation, the larvae were carefully turned to the dorsal side and the time taken to turn back to the ventral side was recorded. Three independent experiments were performed (total *n* = 167).

### 4.7. Larval Crawling

The 3rd instar larvae were collected from both the standard fly food and coconut oil fly food supplementation, washed three times in distilled water, and placed at room temperature on a crawling apparatus. This was a 3% agar plate with a linear agarose channel (2 mm wide, 30 mm long and 5 mm deep), to force the behaviour of the larvae to crawl in a straight and rhythmic manner. After a few minutes of acclimation, each wandering larva was placed on the linear channel and the distance travelled in 30 s and the number of the peristaltic waves were recorded with a graph paper. Three independent experiments were performed (total *n* = 87). 

### 4.8. RNA Isolation and Quantitative Real-Time Polymerase Chain Reaction (qRT-PCR)

Total RNA was extracted from larvae and heads of adult females by using TRI Reagent (Sigma_Aldrich; Merk Life Science S.r.l., 20149 Milano, Italy) according to the manufacturer’s instructions. We considered 3 larvae and 10 fly heads at different stages of disease: presymptomatic (1 day), symptomatic (8 day), and late symptomatic (12 day), per biological replicate. Genomic DNA was digested using a DNase I (Bioline, Meridianlifescience, Italy) digestion protocol, and retrotranscription (1 μg RNA) was performed according to the manufacturer’s instructions (Bioline). Quantitative PCR was performed using SensiFast kit Sybr kit (Bioline, Meridianlifescience, Italy) and the CFX96 Touch Real-Time PCR system. All reactions were carried-out in triplicate and CT values were averaged, normalized to the housekeeping gene *rp49* and compared between groups by using the ΔΔ/Pfaffl method. Primers were designed using Primer3. The sequences used are as follows: RP49Fw AGCTGTCGCACAAATGGC-RP49Rv GTTCGATCCGTAACCGATGT; whdFw CAGGAACTGCAGCCTATCAT-whdRv GCGTGCTGTGTTGAAGGT; Cpt2Fw GGCGAAGAAGATGGAGTCTT-Cpt2Rv TGTCGAACCAACGATTCTG; coltFw GATCTTTGCGATGTGCTTC-coltRv CACGAAGATTTGCGGGTAG; CG6356Fw ATAAGAAGTCGCTGGAGCC-CG6356Rv TTGGAGTTGGCATACAGCT; HNF4Fw GTACACTTGCAGATTTGCGC-HNF4Rv CGCCTTGAAGCACTTCCT; CG10814Fw ACCGGTCATCAACTTGGA CG10814Rv ACCGGCACGCTAAAGTG. 

### 4.9. Statistical Analysis

All data were presented as the mean +/− SEM, and the statistical significance was evaluated using student’s *t* test and one-way ANOVA analysis, followed by a post hoc Sidak’s test for multiple comparisons to determine any statistical differences between groups. Each experiment was performed at least three times. Asterisks were used to indicate a significant difference from the controls. All the data were analysed by the GraphPad Prism v. 7. statistical software package (GraphPad, La Jolla, CA, USA).

## Data Availability

Not applicable.
